# Redistribution of Mitochondria Leads to Bursts of ATP Production During Spontaneous Mouse Oocyte Maturation

**DOI:** 10.1002/jcp.22171

**Published:** 2010-05-04

**Authors:** Yuansong Yu, Remi Dumollard, Andreas Rossbach, F Anthony Lai, Karl Swann

**Affiliations:** 1 Department of Obstetrics and Gynaecology, School of Medicine, Cardiff UniversityHeath Park, Cardiff, UK; 2 Laboratoire de Biologie du Développement UMR 7009 CNRS/Paris VIObservatoire, Station Zoologique, Villefranche sur Mer, France; 3 Cell Signalling Laboratory, Wales Heart Research Institute, School of Medicine, Cardiff UniversityHeath Park, Cardiff, UK

## Abstract

During mammalian oocyte maturation there are marked changes in the distribution of mitochondria that supply the majority of the cellular ATP. Such redistribution of mitochondria is critical for oocyte quality, as oocytes with a poor developmental potential display aberrant mitochondrial distribution and lower ATP levels. Here we have investigated the dynamics of mitochondrial ATP production throughout spontaneous mouse oocyte maturation, using live measurements of cytosolic and mitochondrial ATP levels. We have observed three distinct increases in cytosolic ATP levels temporally associated with discrete events of oocyte maturation. These changes in cytosolic ATP levels are mirrored by changes in mitochondrial ATP levels, suggesting that mitochondrial ATP production is stimulated during oocyte maturation. Strikingly, these changes in ATP levels correlate with the distribution of mitochondria undergoing translocation to the peri-nuclear region and aggregation into clusters. Mitochondrial clustering during oocyte maturation was concomitant with the formation of long cortical microfilaments and could be disrupted by cytochalasin B treatment. Furthermore, the ATP production bursts observed during oocyte maturation were also inhibited by cytochalasin B suggesting that mitochondrial ATP production is stimulated during oocyte maturation by microfilament-driven, sub-cellular targeting of mitochondria. J. Cell. Physiol. 224: 672–680, 2010. © 2010 Wiley-Liss, Inc.

Oocyte maturation in mammals is the final process whereby oocytes acquire their ability to generate an activation response and initiate subsequent embryonic development. This process involves the distinct, although linked, events of nuclear and cytoplasmic maturation (Loutradis et al., 2006; Ferreira et al., 2009). Nuclear maturation involves germinal vesicle breakdown (GVBD), chromosomal condensation and segregation, and polar body extrusion. Cytoplasmic maturation is less characterised but involves organelle reorganization, increases in the content of Ca^2+^ stores, increases in antioxidants (Guerin et al., 2001) and the storage of mRNAs and proteins (Ferreira et al., 2009). Mitochondria play an important role in both of these processes since they provide the main supply of ATP during oocyte maturation (Krisher and Bavister 1998; Stojkovic et al., 2001). Dysfunctional mitochondria and subsequent low ATP production is one of the major factors that compromise oocyte quality (Van Blerkom et al., 1995; Van Blerkom, 2004; Stojkovic et al., 2001; Tamassia et al., 2004; Brevini et al., 2005; Takeuchi et al., 2005; Zeng et al., 2007; Marangos et al., 2008). It has also been proposed that age-related dysfunction in human oocytes could be caused by defective mitochondria (Schon et al., 2000).

The mammalian oocyte typically contains about 100,000 mitochondria that can occupy up to 30% of the cytoplasmic space (Pikó and Matsumoto, 1976; Ankel-Simons and Cummins, 1996; Van Blerkom, 2004; Dumollard et al., 2006). During oocyte growth and the earliest stages of maturation, oocytes are coupled to cumulus cells which provide metabolic support through the provision of ATP and pyruvate (that can be consumed by the oocyte's mitochondria) (Downs 1995; Johnson et al., 2007). However, after ovulation, or during isolation in vitro, mammalian oocytes become uncoupled from surrounding cells and they rely on mitochondria to generate their supplies of ATP (Downs 1995; Dumollard et al., 2006). From ovulation until around the morula stage in mammals, the oocyte, or embryo, relies on mitochondrial oxidative phosphorylation to supply most of the ATP demands for development (Barbehenn et al., 1974; Leese, 1995 review; Dumollard et al., 2006). A significant increase of ATP content in oocytes during maturation has been reported in pig (Brevini et al., 2005) and cattle (Stojkovic et al., 2001), and this ATP increase is correlated with the success rates in embryo development in cattle (Stojkovic et al., 2001), although not in pig (Brevini et al., 2005). ATP content of mature human oocytes is correlated with developmental outcome, and even a transient decrease in ATP during maturation can lead to developmental arrest during cleavage stages (Van Blerkom et al., 1995). These data suggest that the status and activity of mitochondria in the mammalian oocyte is a determining factor of oocyte quality. However, the dynamics of ATP production during the process of oocyte maturation itself has not been reported.

Several studies have examined the morphology and distribution of mitochondria during oocyte maturation or development of embryos (Barnett et al., 1996; Bavister and Squirrell, 2000; Van Blerkom et al., 2000, 2002; Sun et al., 2001; Wilding et al., 2001; Zhang et al., 2008). In the immature oocyte, the mitochondria are aggregated around the germinal vesicle (GV). Once oocyte maturation commences the GV breaks down and the mitochondria are seen to disperse throughout the cytoplasm during maturation, resulting in mitochondria occupying most of the egg volume in a mature MII oocyte (Dumollard et al., 2006). Different variations on this pattern of mitochondrial aggregation occur during oocyte maturation of different species (Van Blerkom and Runner, 1984; Stojkovic et al., 2001; Sun et al., 2001; Wilding et al., 2001; Zhang et al., 2008). This kind of mitochondrial movement is controlled by microtubules, which can directly link to the mitochondria (Heggeness et al., 1978; Van Blerkom, 1991; Nangaku et al., 1994; Sun et al., 2001). Microfilaments are known to play an important role in spindle positioning and cytokinesis, but have so far not been directly linked to mitochondrial movements (Azoury et al., 2009).

It is also known that mitochondria in the oocyte are stimulated at fertilization by the sperm-triggered Ca^2+^ oscillations (Dumollard et al., 2004; Campbell and Swann, 2006). The stimulation of mitochondria is evidenced by changes in mitochondrial NADH and FAD fluorescence (Dumollard et al., 2004). Increased mitochondrial activity at fertilization is also reflected in increased ATP levels detected by imaging the luminescence of firefly luciferase (Campbell et al., 2006; Dumollard et al., 2008). In this study, we have expressed firefly luciferase following cRNA injection into GV stage oocytes, and imaged ATP levels in living oocytes during spontaneous maturation. We have monitored cytosolic and mitochondrial ATP levels by imaging luciferase targeted either to the cytosolic or to the mitochondrial compartment. We show that there is a causal connection between changes in ATP production and mitochondrial clustering and that these changes are directly coordinated by the actin cytoskeleton.

## Results

### The dynamics of ATP during spontaneous oocyte maturation

**Fig. 1:**
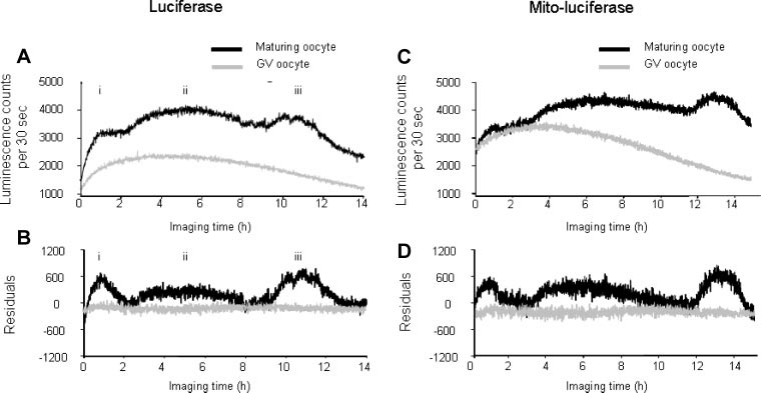
ATP levels during spontaneous oocyte maturation. Relative ATP levels are indicated by the luciferase luminescence in photon counts per 30 sec. A: Three pulses (i, ii and iii) of luminescence in a matured oocyte (dark line) compared to those in a GV arrested oocyte. In order to display these luminescence changes more clearly, the raw data were fitted to a Weilbull curve (B) and the residuals from such a plot were displayed for the same oocytes as in part A. These data are typical of 30 oocytes in three separate replication experiments for maturing and non-maturing oocytes. C: Luminescence from luciferase that is targeted to the mitochondria for a maturing oocyte and a GV arrested oocyte as in part A. Part D: Plot of residuals after fitting a Weilbull curve to the data in (C) and illustrates more clearly the jumps in luminescence. The data in C and D are typical of 30 oocytes. [Color figure can be viewed in the online issue, which is available at http://www.interscience.wiley.com.]

To monitor intracellular ATP levels, we imaged luciferase luminescence expressed in isolated denuded mouse oocytes undergoing spontaneous maturation in vitro. In our initial studies, we found that injection of firefly luciferase protein, as reported by Campbell and Swann (2006), did not result in sufficient luminescence signal for the 12–14-h period of maturation. So instead we injected isolated mouse oocytes with firefly luciferase cRNA before maturation, which allowed for the cumulative expression of luciferase protein over a longer time period. Figure 1A shows typical recordings made from oocytes that underwent GVBD and maturation up to the MII stage. This was compared with control oocytes that were maintained in IBMX to prevent any maturation. A series of three distinct pulses of luminescence are observed in all oocytes that underwent full maturation (the 30 which extruded a polar body) amongst a total of 39 oocytes imaged (Fig. 1A; i, ii and iii on black trace), which contrasts with the smooth curve of luminescence from a non-maturing (IBMX arrested) oocyte (seen in all 17 imaged oocytes, grey trace). Since the curve for the control, non-maturing, oocyte was smooth we found that it could be fitted to a curve. This makes no assumptions about mechanism, but the smooth curve in Figure 1A (grey line) presumably reflects the expression and the eventual destruction of luciferase RNA and protein, respectively (Swann et al., 2009). Since the luminescence from the maturing oocytes showed several distinct deviations from this smooth control curve, the curve fitting process was ineffective. The difference between the fitted curve and the observed luminescence is represented as the residuals (Fig. 1B,D). Figure 1B shows the residuals plotted from the curve fit and it clearly illustrates the three distinct pulses of luminescence from an oocyte undergoing maturation (black line) compared with the flat line (grey line in Fig. 1B) of residuals from an oocyte that did not undergo maturation. These changes in luminescence did not appear to be due to an imaging artefact because when we injected 70 kDa fluorescein dextran (which has a similar molecular weight to luciferase) into maturing oocytes and recorded fluorescence for 14 h and we observed no significant changes in intensity (Supplementary Fig. 1). Fluorescence is more prone to artefacts caused by changes in distribution of a probe, or changes in shape of a cell. Consequently, these data suggest that the changes in luminescence occur because there are three phases of increased ATP levels occurring during spontaneous maturation of mouse oocytes.

We have previously shown that mitochondrial ATP can be monitored in mouse oocytes with a firefly luciferase targeted to the mitochondria (Dumollard et al., 2008). So we also injected mito-luciferase cRNA and monitored luminescence in maturing oocytes. Figure 1C shows that the mitochondrial luminescence also exhibited the distinct increases in ATP in oocytes undergoing maturation (black line). The luminescence changes can be discerned from the raw plot, or from the residuals plotted after curve fitting (Fig. 1D). All 22 control oocytes injected with mito-luciferase and held at GV by the presence of IBMX failed to show any distinct transient rises in luminescence (grey lines in Fig. 1C,D). Similar changes in mitochondrial luciferase luminescence were observed in all 30 oocytes that underwent maturation, suggesting that the phases of increased cytosolic ATP levels are linked to three phases of mitochondrial ATP production. The data in Figure 1 therefore suggest that there are three pulses of ATP generation during spontaneous mouse oocyte maturation and that these are due to the stimulation of mitochondrial activity.

### Mitochondrial remodelling during maturation

**Fig. 2:**
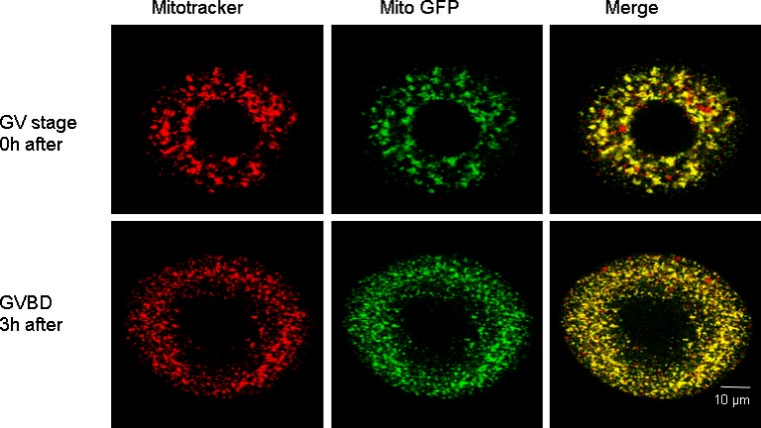
The distribution and morphology of mitochondria in immature oocytes at the GV stage, and 3 h after release from IBMX at which point GV breakdown (GVBD) had occurred. In the top row, mitochondria with an intact GV were labelled with mitotracker orange and mito-GFP. In the bottom row an oocyte was similarly treated after GV breakdown (GVBD). The merged images show that, in both types of oocytes, the mitotracker staining pattern matches that labelled with mito-GFP.

To investigate factors that might stimulate mitochondria, we decided to examine their distribution. Mitochondrial translocation and aggregation have been shown to occur during oocyte maturation, but these patterns have only been described at a limited number of time points (Van Blerkom and Runner, 1984; Stojkovic et al., 2001; Sun et al., 2001; Wilding et al., 2001; Zhang et al., 2008). Moreover, these studies address rather the general distribution of mitochondria whereas mitochondrial clustering is poorly described. We stained oocytes with mitotracker-orange and observed them every hour for the first 10 h of maturation. The specificity of labelling was first verified by comparing mitotracker orange staining with mito-GFP (Aida et al., 2001). Figure 2 shows the staining pattern with these two probes in a GV oocyte, and in an oocyte that had undergone GVBD. There is a good correlation between the two staining patterns, with only the occasional larger round vesicle being stained with mitotracker and not mito-GFP. The overall distribution and pattern of staining is sufficiently well correlated in both types of oocytes to make observations on the distribution of mitochondria in oocytes using mitotracker labelling, which is a rapid and simple procedure.

**Fig. 3:**
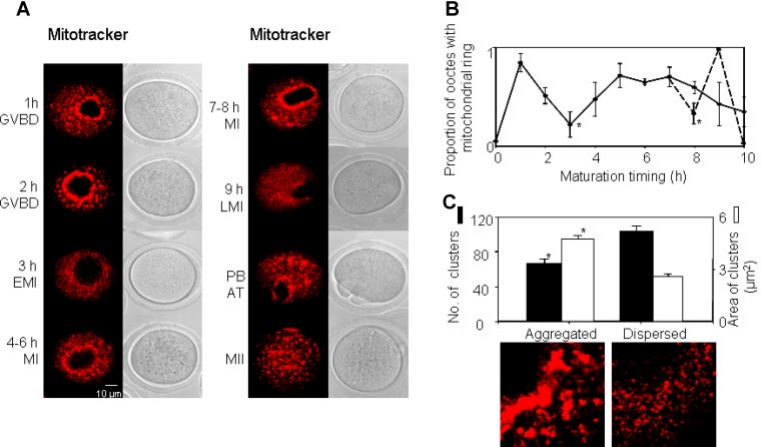
Mitochondria patterns during maturation. In GV oocytes, mitochondria distribute in the whole oocyte, with some aggregation around the GV area (as shown in Fig. 2). Part A shows that the peri-nuclear/spindle mito-rings form soon after release from inhibition, but disappears at early MI (EMI) stage (around 3 h after the start of maturation) and in the late MI (LMI) stage (around 9 h into maturation). The ring is also present at MI (4–8 h after the start of maturation) and the polar body extruding stage (9–10 h, PB anaphase-telophase stage). Part B shows a plot generated from all oocytes (n = 336) of the dynamics of mitochondrial ring formation during maturation. The solid line represents the proportion of mitochondrial rings in oocytes at different time points. The dashed line shows mitochondrial rings in GVBD oocytes, polar body extruding oocytes, and matured MII oocytes among all oocytes collected at 8, 9 and 10 h after the start of maturation. Part C shows the differences between two patterns of mitochondrial classified according to the size and number. The mitochondrial pattern at 1, 2, 4–6, 7–8 h, AT, and MII stages was classified as ‘aggregated’ (left image in Part C, taken from image of 2 h in Part A) and had larger sized mitochondria that were less in number than in the ‘dispersed’ pattern (right image in Part C, taken from image of 9 h/LMI in Part A) which was seen in oocytes at 3 and 9 h after the start of maturation. [Color figure can be viewed in the online issue, which is available at http://www.interscience.wiley.com.]

Figure 3 shows that, when GVBD starts, mitochondria translocate from the outer cytoplasm to the perinuclear area, as described previously (Dumollard et al., 2006). A striking, dense mitochondrial ring (mito-ring) around the GV area can be clearly seen as soon as the GVBD occurred (which was about 1 h after removal from IBMX, Fig. 3A). It is noteworthy that whilst most of the GV stage oocytes show some mitochondria surrounding the GV, these were not scored as having a mitochondrial ring, because they were not as dense as the ring formed at GVBD. Interestingly, the early stage perinuclear mitochondrial ring that we observed, dispersed at the early stage of the first metaphase (EMI) around 3 h after release from IBMX. At 4–6 h post-IBMX release when most oocytes entered first metaphase (MI), a new mitochondrial ring formed around the area predicted to be the spindle. This secondary mitochondrial ring gradually elongated with the spindle rearrangement (MI, Fig. 3A, 7–8 h). When oocytes started extruding a polar body, there was always a clear mitochondrial ring around the spindle (100% 7/7, Fig. 3A, ‘PB’). However, since this stage of maturation was not synchronous for all oocytes, this third mitochondrial aggregation phase was not evident in the graph illustrating proportions of different mitochondrial patterns versus time in a population of maturing oocytes (solid line graph in Fig. 3B). Hence, we re-plotted the data for the final 8–10 h stages of maturation, grouping oocytes as either belonging to those prior to first polar body emission (LMI), those undergoing first polar body emission or those having reached MII. In this case, the dashed line (Fig. 3B) shows that there is a third phase of mitochondrial translocation in oocytes undergoing polar body extrusion. The mitochondrial ring disappeared in MII stage oocytes (Fig. 3A, MII).

The temporal changes shown in Figure 3 are reminiscent of the bursts of ATP production (Fig. 1), suggesting that the formation of a peri-nuclear mitochondrial ring could play a role in stimulation of mitochondrial activity. However, there was another change in mitochondrial distribution that was seen in maturing oocytes. This involved a change in the size of mitochondrial clusters. At GVBD, only 5% of oocytes show small mitochondrial clusters (on average 2.6 ± 0.13 µm^2^) whereas most mitochondrial clusters are larger (on average 4.7 ± 0.2 µm^2^, *P* < 0.01). In contrast, at 3h and at 9h after release from IBMX, a large proportion of oocytes (39.2 ± 12.3% and 42.9 ± 4.9% vs. 3.7 ± 3.7% (at 1 h) and 16.7 ± 10.3% (at 5 h) respectively, *P* < 0.05) contain mitochondria that occur in smaller clusters throughout the cytoplasm (Fig. 3A,C). Consistent with mitochondrial aggregates size, less mitochondrial rings formed at these two stages (17.9 ± 6.8% or 31.6 ± 11.3% (LMI) vs. 83.8% ± 4.3% (at 1 h) or 70.1 ± 6.5% (at 5 h). respectively, *P* < 0.05). At these two stages there also appears to be a larger number of mitochondrial clusters (102.4 ± 5.9/per equatorial section). We refer to these mitochondria as having a dispersed pattern and this pattern correlates with times of relatively low ATP production. However, at stages of higher ATP production (1h, 5h after IBMX release, and at the polar body extrusion stage), most oocytes (96.3 ± 16.5%, 83.3 ± 7.1% or 100% vs. 60.8 ± 10.6% (at 3 h) or 57.1 ± 4.9% (at 9 h) respectively, *P* < 0.05) showed an aggregated pattern of mitochondria. This was evident as an apparently lower number (66.2 ± 4.7/per equatorial section, *P* < 0.01) of mitochondrial clusters of larger size (Fig. 3C). These data show that the changes in ATP levels we observed are temporally correlated with changes in the clustering pattern of mitochondria, suggesting that these changes may be a factor in mitochondrial ATP production stimulation.

### Role of the cytoskeleton in the regulation of mitochondrial distribution and ATP changes

**Fig. 4:**
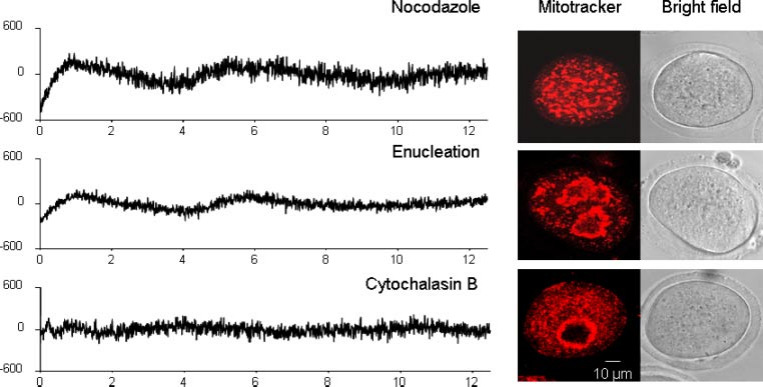
The effect of cytoskeleton or enucleation on the ATP dynamic during maturation. All graphs of ATP changes were generated from regression residual data as described in Figure 1. Both enucleation (n = 12) and nocodazole (10 µM, n = 31) completely removed the third ATP jump that occurs around 8–10 h after release from IBMX inhibition, but did not affect the first two jumps of ATP. However, ATP jumps in most of oocytes (75 ± 4.8%, 27/36) treated with cytochalasin B (10 µg/ml) were greatly reduced or abolished. In contrast to nocodazole or enucleation treatment, a smaller third ATP jump was seen in some cytochalasin treated oocytes (14/36). The right part of photos showed that both enucleation and cytochalsin B treatment did not affect the translocation to the nuclear area, but nocodazole removed the mitochondrial peri-nuclear/spindle ring. [Color figure can be viewed in the online issue, which is available at http://www.interscience.wiley.com.]

Previous studies showed that mitochondrial movements in mouse oocytes were microtubule dependent (Van Blerkom, 1991). In order to establish whether the ATP changes observed during maturation were caused by mitochondrial translocation, oocytes were treated with 10 µM nocodazole before and during the whole the maturation period. Nocodazole treatment did not block the formation of large mitochondrial clusters, whereas the perinuclear ring of mitochondria vanishes (together with the meiotic spindle) (Fig. 4, right top part). Nocodazole also blocks the completion of metaphase I and polar body extrusion through activation of spindle assembly checkpoint (Wasserman et al., 2003). Strikingly, oocytes maturing in the presence of nocodazole displayed the first two bursts of ATP production, but not the third one (93.5 ± 2.3% (29/31), Fig. 4). Enucleation, a technique which removes the DNA/chromosomes but not the perinuclear/perispindle mitochondrial ring, also ablates the third burst of ATP production (100% (12/12), Fig. 4). These observations demonstrate that the third burst of ATP production is strictly associated with the completion of meiosis I (and PB extrusion). In contrast to nocodazole, cytochalasin B treatment (10 µg/ml) throughout the period of maturation either diminished or completely removed the three ATP transients in the majority of oocytes (Fig. 4, cytochalasin B, 75 ± 4.8% vs. 0 in control oocytes *P* < 0.01). We noticed that a small third increase occurred in some oocytes, but the first two ATP changes were not observed (data not shown). We also investigated the effects of latrunculin A, which also disrupts actin filaments. In the presence of latrunculin A oocytes also failed to show any of the jump like changes in fluorescence (see Supplementary Fig. 2). However, the absolute level of luminescence from oocytes in the presence of latrunculin A was lower than control oocytes suggesting an effect upon protein expression, or upon ATP generation. In contrast the absolute level of luciferase luminescence in cytochalasin B experiments was similar to control oocytes, suggesting a lack of effect of this drug upon resting ATP levels. The observations with cytochalasin B are, therefore consistent with the first two increases in ATP production being dependent upon microfilament dependent events.

**Fig. 5:**
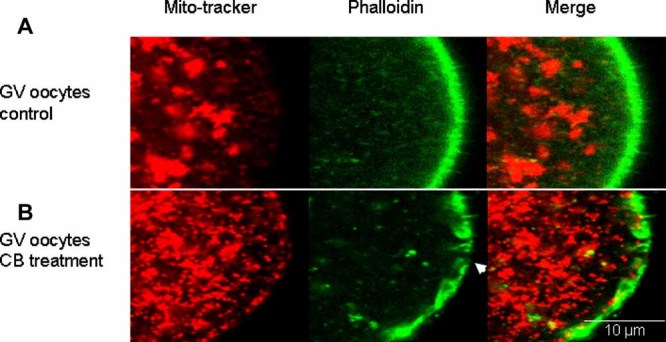
The effects of cytochalasin B treatment on the mitochondrial clusters and microfilaments. Mitochondria and microfilaments in GV oocytes were stained with mitotracker-orange (red) and alexa-phalloidin 488 (green) respectively. In (A) the GV oocyte's mitochondria were present in large clusters and F-actin was concentrated under the plasma membrane. In (B) the effects of cytochalasin B treatment can be seen in a GV oocyte where the mitochondria are dispersed into smaller fragments and the microfilaments have developed clefts beneath the plasma membrane.

The above data show that the formation and dissolution of the mitochondrial ring is not by itself the key factor in determining the first two bursts of ATP production because nocodazole abolishes the ring but not the first two changes in ATP, and cytochalasin B inhibits the changes in ATP but not the formation of a mitochondrial ring. In contrast the data suggest that the first tow bursts of ATP production rely on microfilaments which could be effecting the reorganisation of mitochondrial clusters. We therefore examined mitochondria and actin filaments in more detail during oocyte maturation. Microfilaments in oocytes were stained with Alexa 488-phalloidin. Figure 5 shows that GV oocytes contained mitochondria distributed in large clusters in the cytoplasm with the microfilaments nucleated under plasma membrane (cortical area) (Fig. 5A). Treatment of GV-blocked oocytes with cytochalasin B for several hours resulted in oocytes with more dispersed mitochondrial clusters and it caused clefts in the microfilament signal ring under membrane (Fig. 5B, arrow points to the cleft). Because large mitochondrial clusters are already present in GV oocytes, it can be inferred that cytochalasin B disperses large clusters of mitochondria into smaller clusters. Since it also blocks the early increases in ATP production, these data imply that large mitochondrial clusters are underlying the generation of the first two bursts of ATP production.

### The remodelling of microfilaments and mitochondria during oocyte maturation

**Fig. 6:**
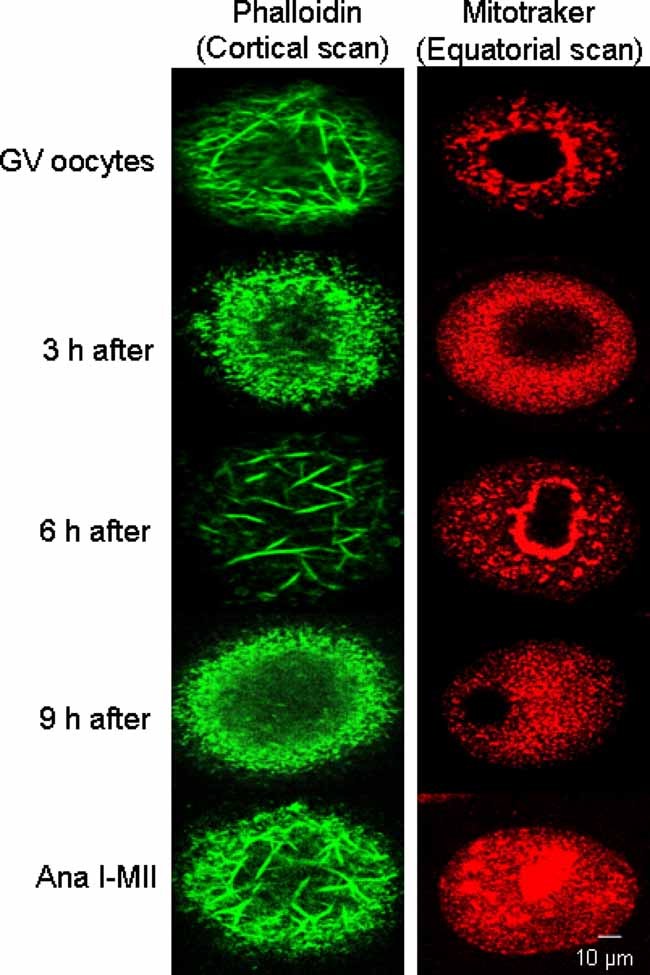
The cortical microfilament network and mitochondria during oocyte maturation. The left part (green) shows oocytes stained with alexa-phalloidin and the right part shows oocytes stained with mitotracker orange as in previous slides. The images are collected from oocytes before, and at different times after the start of maturation (times indicated in the left hand side of each pair of images). In GV oocytes, large patches of mitochondria and a dense microfilament network can be seen. A breakdown of mitochondria clusters and cortical microfilaments occurs at 3 h after the start of maturation when GV breakdown had occurred. However, larger mitochondrial clusters and dense cortical microfilaments re-appeared 6 h after release. A further dispersion of mitochondria and microfilaments then re-occurs at 9 h after the start of maturation. [Color figure can be viewed in the online issue, which is available at http://www.interscience.wiley.com.]

The above data on the effects of cytochalasin B imply that the microfilament network is a key organizer of cytoplasmic maturation. To examine this idea we monitored the distribution of microfilaments and mitochondria throughout spontaneous oocyte maturation. Figure 6 shows a GV oocyte that possesses both a network of cortical microfilaments and large mitochondrial clusters. Just after GVBD (3 h after IBMX wash), cortical actin filaments are disrupted. However, the microfilament network eventually reformed and, by 6 h (during migration of MI spindle) cortical actin filaments can be seen. The microfilament network collapsed again at ∼9 h (at the end of spindle migration) synchronous with mitochondrial clusters being re-dispersed. Finally, during PB extrusion (anaI-MII) and later (MII mature oocytes), dense microfilaments and large mitochondrial clusters formed again. These data show a strong correlation between the pattern of mitochondrial clusters and cortical actin network during maturation. As cytochalasin B dispersed mitchondrial clusters, our observations suggest that remodelling of the actin network underlies the reorganization of mitochondrial clustering resulting in the observed bursts of ATP production.

## Discussion

**Fig. 7:**
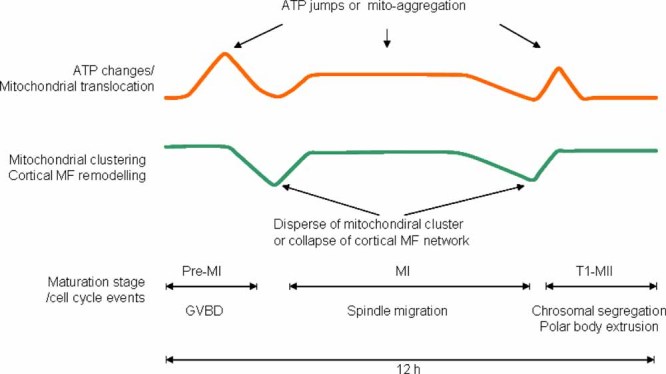
A schematic diagram to show the proposed timing of ATP changes, mitochondrial translocation and clustering, cortical microfilament (MF) remodelling and cell cycle changes, during mouse oocyte maturation. All three bursts of ATP production occur when there is a dense cortical microfilament network, and larger clusters of mitochondria. [Color figure can be viewed in the online issue, which is available at http://www.interscience.wiley.com.]

This study is the first report of ATP dynamics in a living oocyte of any species during maturation. The data presented suggest that there are changes in the rate of mitochondrial ATP production during spontaneous mouse oocyte maturation. In particular, there are three distinct increases in cytosolic and mitochondrial ATP levels indicative of three phases of higher ATP production, interspersed between two phases of lower ATP production. The first phase of increased ATP production occurs around the time of GV breakdown (1–2 h after release from IBMX). The second occurs during the longer phase of spindle migration (between 5 and 7 h) and the third phase is during the MI to MII transition (from 11 to 12 h, see Fig. 7). Whilst we may not expect that the increases in ATP have a direct function on the cell physiology, it is quite likely that they are a measure of events that are occur in energy metabolism during maturation. In fact our analysis of mitochondrial distribution reveals a close temporal correlation between the changes in ATP production and the aggregation patterns of mitochondria, which are causally linked to discrete changes in microfilament organization. To our knowledge, this investigation is the first time microfilaments, as opposed to microtubules, have been directly implicated in mitochondrial function or distribution in oocytes.

### Maturation is accompanied by dynamic changes in ATP levels

We have previously used the luminescence of firefly luciferase to monitor ATP changes at fertilization in both the cytosol and mitochondria of mouse oocytes (Dumollard et al., 2004; Campbell and Swann, 2006; Dumollard et al., 2008). Live imaging of firefly luciferase luminescence has also been shown to effectively monitor ATP in muscle and pancreatic beta cells (Allue et al., 1996; Kennedy et al., 1999). We found that injection of cRNA was required for the relatively long recording times of oocyte maturation. We previously found that injection of luciferase cRNA into mature mouse oocytes gives rise to a luminescence signal that follows a smooth curve, reflecting initial expression and then destruction of luciferase protein (Swann et al., 2009). Such a smooth curve of luminescence is also evident in mouse oocytes injected with luciferase cRNA and held at the GV stage for 10 h. The actively maturing oocytes, however, clearly shows three distinct phases of deviations from the smooth curve of luciferase expression and destruction. The ATP sensitivity of firefly luciferase indicates that these deviations are due to ATP changes. Since luciferase targeted to the mitochondria showed the same pattern of deviation, the data imply that it is the mitochondria specifically generating extra ATP during three distinct phases of oocyte maturation. Furthermore, there were no jump like changes in fluorescence from fluorescein dextran recorded under the same conditions of oocyte maturation (Supplementary Fig. 1) so its seems unlikely that the changes are generated by an imaging artefact since such single wavelength fluorescence is more prone to such issues than luminescence. The only other potential cause of such positive deviations in luciferase luminescence could be changes in intracellular pH (Allue et al., 1996). However, there are no indications that pH changes during maturation of fully grown mouse oocytes (Fitzharris and Baltz, 2009). Furthermore, it is unlikely that a mitochondrial luciferase would respond to any potential cytosolic pH change. Hence, the most likely cause of the luminescence pulses shown in Figure 1 are that there are three pulses of ATP generation caused by increased activity of mitochondria.

### Reorganisation of mitochondria during oocyte maturation

To identify the potential causes of changes in mitochondrial activity we examined their distribution during oocyte maturation. Mitochondrial translocation to the area around the GV in oocytes has been reported previously (Van Blerkom and Runner, 1984; Calarco, 1995; Stojkovic et al., 2001; Sun et al., 2001; Wilding et al., 2001; Zhang et al., 2008). During in vitro maturation we found that the mitochondrial translocation and aggregation around GV or spindle area occurred repeatedly. The first peri-nuclear/spindle mitochondrial-ring forms immediately after IBMX release and this reaches a peak 1 h later at GVBD. The second mitochondrial ring lasts for several hours from 5 to 7 h after IBMX release, while the third one is limited to the time of first polar body extrusion. However, these localizations around the nucleus do not appear to be significant in terms of ATP generation since blocking the perinuclear localization of mitochondria with nocodazole did not block the first two pulses of ATP during maturation. Significantly, we observed a more subtle change in the localization of mitochondria on a smaller scale. Mitochondrial clustering within the cytoplasm has been described in mouse oocytes (Calarco, 1995), however we have discovered that the clustering pattern correlates with the timing of ATP pulses. Specifically, it appeared that the formation of large clusters of mitochondria is associated with increased ATP production. Unlike perinuclear aggregation the clustering of mitochondria appears to be causal because cytochalasin B treatment both prevents the formation of clusters of mitochondria and inhibits the bursts of ATP production.

Previous studies have suggested that mitochondria movement in oocytes was not dependent upon microfilaments (Van Blerkom, 1991; Sun et al., 2001). However, mitochondria can be in a close association with microfilaments in somatic cells (Morris and Hollenbeck, 1995). Furthermore actin-–myosin interactions underlie mitochondrial organization in yeast (Drubin et al., 1993). Microfilaments are also important for normal mitochondrial axonal transport in neurons (Morris and Hollenbeck, 1995). Although we have not shown a direct link between mitochondria and cytoplasmic microfilaments, a close relationship was seen between mitochondrial clustering and cortical microfilament remodelling. A recent study (Azoury et al., 2008) has shown a reorganization of actin filaments in the oocyte cortex during polar body extrusion, and this kind of reorganization reflects a translocation of actin filament between cortex and cytoplasm (Terasaki, 1996).

Our study reveals that the formation of a microfilament network during GVBD entrains mitochondrial clustering, resulting in improved mitochondrial ATP production at GVBD. The microfilament network disassembles, entraining mitochondrial cluster dispersion and a drop in ATP production. This cycle of microfilament-driven mitochondrial clustering and dispersion occurs a second time during spindle migration. The mature oocyte possesses a long microfilament network and large mitochondrial clusters to be ready for fertilization, during which ATP production is further increased by sperm-triggered Ca^2+^ signals (Dumollard et al., 2006). It is not clear why the degree of clustering of mitochondria affects their ability to generate ATP. At fertilization, the mitochondria in the oocyte are stimulated by Ca^2+^ oscillations in the cytoplasm which cause mitochondrial Ca^2+^ increases (Campbell and Swann 2006; Dumollard et al., 2006). However, the only changes in Ca^2+^ during mouse oocyte maturation consist of a series of small oscillations that occur within the first 2 h of release from follicles (Carroll and Swann, 1992). These Ca^2+^ oscillations would have stopped before the start of our experiments since oocytes are incubated for 3 h in IBMX before measuring luciferase luminescence. As there are no further Ca^2+^ changes later during oocyte maturation (Marangos and Carroll, 2004), it seems unlikely that Ca^2+^ plays any role in stimulating ATP changes in a maturing oocyte. The mechanism linking mitochondrial clustering and mitochondrial ATP production remains to be elucidated.

## Materials and Methods

### Oocytes collection and in vitro maturation

Female ICR mice were primed with 7.5 IU pregnant mare serum gonadotrophin, and killed by cervical dissociation 48 h later. Ovaries were dissected and follicles punctured with needles in M2 medium (Sigma, Poole, Dorset) containing 0.5 mM 3-isobutyl-1-methyl xanthine (IBMX, Sigma). Oocytes surrounded by cumulus cells were collected and denuded manually with fine bore glass pipettes. All oocytes were kept in M2 medium containing IBMX prior to maturation. Oocytes were allowed to mature by washing into drops of M2 medium, without IBMX, that were at 37°C, either in a 5% CO_2_ incubator or on a heated stage of the imaging microscope.

### Micromanipulation of oocytes

Oocytes were microinjected with 3–5% of the oocyte volume of a solution containing different concentrations of firefly luciferase-cRNA (1 µg/µl), or mitochondrially-targeted firefly luciferase (mito-luciferase) cRNA (1 µg/µl), or mitochondrially-targeted GFP (mito-GFP) cRNA (1 µg/µl), or firefly luciferase protein (5 mg/ml, Sigma) or alexa 488-phalloidin (2 µM, Invitrogen Ltd., Paisley, UK). The mito-GFP and mito-luciferase constructs were cloned into pRN3 vectors, and the firefly luciferase construct was cloned into a pcDNA3 vector. Further details on the preparation of these constructs are given in Dumollard et al. (2008). Complementary RNA for each construct was prepared with mMessage mMachine T3 and T7 kits (Applied Biosystems, Warrington, UK) respectively, followed by polyadenylation (Aida et al., 2001).

Some GV stage oocytes were enucleated as described before (Greda et al., 2006). Briefly, a pipette with a tip of 1–2 µm, was pushed through the zona to make contact with both the plasma membrane and the GV membrane. Then negative pressure was applied to ensure that a small part of the GV membrane was strongly held by the pipette. Then the pipette was slowly pull out of oocyte and zona during which the GV was broken. Finally, the GV membrane was completely pulled out of the oocyte leaving nuclear material inside.

### Dynamic ATP measurements

In order to image the dynamics of ATP level in oocytes during maturation, luciferase-cRNA, or mito-luciferase cRNA were injected into GV stage oocytes that were then maintained in M2 medium containing IBMX, with or without treatments (10 µM nocodazole or 10 µg/ml cytochalasin B) for 3 h. Oocytes were then washed out of IBMX and placed in M2 medium containing 100 µM luciferin (Sigma) (and cytochalasin B or nocodazole as required) on the heated stage of a microscope equipped with a photon counting imaging camera based on an intensified CCD (Photek Ltd., St Leonards on sea, UK), as described in Campbell and Swann (2006). Luminescence signals reporting ATP level in oocytes were imaged for 13–16 h. Plots of luminescence were made as photon counts per 30 sec. However, in order to display more clearly the changes of ATP levels, a regression peak analysis was carried out by fitting a Weibull or log normal plot to the data and then plots were generated from the residuals from the fitted curve.

### Mitochondrial aggregate quantification

According to the staining of mitochondria with mitotracker, the diameter of one individual mitochondria is about 0.5–1 µm, Hence the area for one mitochondria is about 0.2–0.8 µm^2^. We used ImageJ (http://rsbweb.nih.gov/ij/) to quantify the number or the size of mitochondrial clusters in classified oocytes. The images of mitochondria were initially adjusted to be distinct by threshold setting. We then used the ‘Analyse Particles’ function to automatically count the number and area of aggregates. The significance of the differences in the number or area of aggregates was analysed using a unpaired *t*-test.

### Mitochondrial or microfilament staining and observation

Mitochondrial staining was carried out by incubating oocytes in M2 medium containing 0.5 µM mito-tracker orange (Invitrogen Ltd.) for about 20–30 min immediately prior to image collection. Microfilaments were labelled by injecting alexa 488-phalloidin (Invitrogen Ltd.) which was dissolved in methanol at a concentration 6.6 µM. Before injection, 10 µl of the phalloidin solution was dried down in nitrogen to about 2 µl and then re-dissolved in 10 µl of Hepes-buffered KCl solution (Terasaki, 1996). All the observations of mitochondria or microfilaments were made using a confocal microscope (TCS SP5; Leica, Milton Keynes, UK), and a 60× oil immersion objective lens. The excitation laser for mito-tracker orange, mito-GFP or alexa 488-phalloidin were 561, 488 and 514 nm, respectively. Equatorial or submembrane (3–4 µm from surface) section scanning was performed with a fixed pinhole size (1.5 µm), and a sequential scanning mode was adopted if the eggs were stained with both mito-tracker orange and Alexa 488-phalloidin at the same time. Confocal images were analysed and edited using ImageJ (http://rsbweb.nih.gov/ij/).
