# Supplementary material for: Redistribution of Mitochondria Leads to Bursts of ATP Production During Spontaneous Mouse Oocyte Maturation
Source: J Cell Physiol. 2010 May 4;224(3):672–80. doi: 10.1002/jcp.22171 (PMC3149123; doi:10.1002/jcp.22171)
Supplement: Supplementary file 1 [file jcp0224-0672-SD1.doc]

Supplementary Fig. 1. The dynamics of control fluorescence during oocytes maturation. Freshly collected GV oocytes were injected with 2mg/ml FITC-dextran (MW 70,000) and kept in M2 medium with IBMX for 3 h before imaging to mimic the protocol used in luciferase imaging. The dynamics of fluorescence intensity (photon counts per 10 sec) during oocyte maturation is plotted in A, with the residuals plotted in B (as for Fig. 1).

Supplementary Fig. 2. The effect of latrunculin A and cytochalasin B treatment on the luciferase luminescence in maturing oocytes. The luminescence fails to show major pulses in cytochalasin B treated oocytes (left panel) but the overall luminescence is similar to control oocytes shown in Fig. 1. In oocytes treated with latrunculin A, there are also no extra pulses in luminescence but the absolute level of luminescence was much lower (peak <1000 cps in all 25 oocytes) than in cytochalasin B treated or control oocytes.
